# INAM-Based Image-Adaptive 3D LUTs for Underwater Image Enhancement

**DOI:** 10.3390/s23042169

**Published:** 2023-02-15

**Authors:** Xiao Xiao, Xingzhi Gao, Yilong Hui, Zhiling Jin, Hongyu Zhao

**Affiliations:** 1 State Key Laboratory of CEMEE, Luoyang 471000, China; 2 School of Telecommunications Engineering, Xidian University, Xi’an 710071, China; 3 Science and Technology on Complex System Control and Intelligent Agent Cooperation Laboratory Beijing Electro-Mechanical Engineering Institute, Beijing 100074, China

**Keywords:** image enhancement, underwater images, instance normalization

## Abstract

To the best of our knowledge, applying adaptive three-dimensional lookup tables (3D LUTs) to underwater image enhancement is an unprecedented attempt. It can achieve excellent enhancement results compared to some other methods. However, in the image weight prediction process, the model uses the normalization method of Instance Normalization, which will significantly reduce the standard deviation of the features, thus degrading the performance of the network. To address this issue, we propose an Instance Normalization Adaptive Modulator (INAM) that amplifies the pixel bias by adaptively predicting modulation factors and introduce the INAM into the learning image-adaptive 3D LUTs for underwater image enhancement. The bias amplification strategy in INAM makes the edge information in the features more distinguishable. Therefore, the adaptive 3D LUTs with INAM can substantially improve the performance on underwater image enhancement. Extensive experiments are undertaken to demonstrate the effectiveness of the proposed method.

## 1. Introduction

### 1.1. Background

In recent years, with the excavation and exploration of marine resources and the ocean world, high-quality underwater images have become increasingly important. The processing and enhancement of underwater signals and images have also attracted a lot of attention. However, the complex underwater environment and lighting conditions significantly pose great challenges for underwater image enhancement, which aims to improve the image visibility and contrast and reduce chromatic aberration. The reasons are as follows. Firstly, underwater images will be affected by noise from marine snow, which increases the scattering effect and dramatically reduces the contrast and visibility of the image. Secondly, underwater images are degraded by wavelength-dependent absorption and scattering including forward and back scattering [1,2,3,4,5], which all limit the practical application of underwater images and videos in marine biology, archaeology, marine ecology, and ocean exploration. In terms of performance and efficiency, underwater image enhancement remains a significant challenge due to the diversity of captured scenes, the complexity of underwater environments, and the fluctuation of underwater lighting conditions.

### 1.2. Related Works

To solve the above problems, a number of methods have been proposed. (1) Supplementary information-based methods. The method proposed by Narasimhan et al. [6] is to use the supplementary information of multiple images to increase the visibility of the image. Some methods [7,8,9,10] use special hardware such as polarization filtering to improve the image visibility. ERH [11] uses three procedures for color compensation, image alignment, and homogenization using a multiscale synthesis strategy. The ERH method cascades the three procedures for underwater image enhancement. (2) Non-physical model-based methods. The method proposed by Iqbal et al. [12] modifies the image pixel values in RGB (Red, Green, Blue) color space and HSV (Hue, Saturation, Value) color space through Non-Physical Model-Based Methods to improve the contrast and saturation of underwater images. (3) Physical model-based methods. Underwater image enhancement is carried out through Physical Model-Based Methods [13]. This method sets a cost function and increases the image contrast by reducing the cost function, thereby obtaining excellent underwater images. GUDCP [14] is a new method for backward scattered light estimation that integrates several prior knowledge and introduces a new scoring formula. This method also develops a white balance method to further modify the appearance of the synthetic image. There are also excellent methods such as SVM-RBF [15] in remote sensing that provide inspiration for underwater image processing.

These years, many deep learning-based image enhancement methods [16,17,18,19,20,21,22] have been proposed in the field of computational imaging. MFFN [23] is a multi-scale feature fusion network that can enhance the adaptability and visualization of the scene. SGUIE-Net [24] is a semantic region-based enhancement module that can better learn local enhancement features of semantic regions with multi-scale perception. The fused features are semantically consistent and visually have better enhancement effects. The AGA-based Swin Transformer module [25] is designed to be an end-to-end underwater image enhancement network. It can dynamically select visually complementary channels based on dependencies, reducing the number of further attention parameters. R. Liu et al. developed a bilaterally constrained closed-loop adversarial enhancement module [26] that alleviates the requirements of the unsupervised approach for pairwise data by coupling twin inverse mappings and preserves more informative features. J. Yuan et al. proposed a multi-scale fusion enhancement algorithm [27] to improve sharpness by contrast-based a priori de-fogging of the dark channel in the red-green-blue (RGB) model. However, some of them have complex network structures or expensive computational cost. Zeng, H et al. proposed the learning image-adaptive 3D LUTs (three-dimensional lookup tables) hybrid method [28], combining multi-layer features in deep learning-based methods and image priors in traditional methods. This image enhancement method has high performance, high image quality, high computational efficiency, and low memory consumption. However, the normalization method of instance normalization adopted by the CNN (Convolutional Neural Network)-based weight predictor in this method will lead to the degradation on the network performance. Instance Normalization (IN) [29] is a milestone technique in deep learning that normalizes the distribution of intermediate layers, leading to faster training and better generalization accuracy. However, the residual features’ standard deviation is greatly reduced after normalization. The standard deviation reflects the variation of pixel values. When the variation is reduced, the ability of the network distinguishing edges will decrease, further degrading the network performance, which significantly impacts on the performance of underwater image enhancement methods.

### 1.3. Contributions

In this paper, we propose an Instance Normalization Adaptive Modulator (INAM) that amplifies pixel bias by adaptively predicting modulation factors to solve this issue. The Instance Normalization operation applies normalization to a specific image instance and normalizes it in the HW (Height and width) dimension of the tensor, and is initially used for image style transfer. The mean and variance of each channel of the feature map affect the style of the final generated image. We introduce INAM into the learning image-adaptive 3D LUTs for underwater image enhancement. The bias amplification strategy in INAM makes the edge information in the features more distinguishable.

The environmental and lighting conditions underwater lead to poor visibility, low contrast, and large color differences from natural scenes in underwater images. 3D LUT itself is an artificially produced method for image enhancement. Adaptive 3D LUT, on the other hand, can be learned by a deep learning method driven by data, which compares the input poor visibility image with clear ground truth. In this process, the weight prediction of the image is critical. The weights not only determine the weight of the LUT’s influence on the image but also affect the parameter learning of the 3D LUT itself under adaptive conditions. Our proposed INAM can effectively improve the problem caused by IN in CNN by amplifying the pixel bias and making the network more capable of discriminating the edges of objects in images. The introduction of INAM enables CNN to predict the weights of images more accurately and further affects the parameters learned by 3D LUTs. It gives the adaptive 3D LUTs a more vital ability to enhance the images. In contrast to other methods, we combine traditional lookup tables and deep learning methods. We exploit the powerful learning ability of deep learning models, which makes manual lookup tables unnecessary, dramatically reduces the workload, and has high accuracy and precision. Our proposed INAM compensates for the IN layer drawback of the weight prediction module, which enables a more efficient and accurate combination of weight prediction and image enhancement. Our model has low complexity, a small number of parameters, and uses only a few computational resources to achieve good enhancement results.

Our approach breaks the limits of deep learning and combines it with traditional methods. It also uses deep learning to simplify the complex work of traditional methods greatly. Significantly, our proposed INAM also reduces the drawbacks of IN, which allows CNNs to ignore the effects of residual feature reduction in weight assignment, substantially improving the edge information recognition and the accuracy of weight assignment. Our method provides a new idea for underwater image enhancement.

The main contributions of the work are summarized as follows:

(1) We propose INAM, which enhances CNN recognition of image edge information by adaptive modulation factors to amplify pixel deviations. Compared with current methods, this module has few parameters, and substantial performance improvement on CNN with almost no increase in computational resources.

(2) We introduce INAM into adaptive 3D LUTs to form adaptive 3D LUTs and train them for data-driven deep learning. We improve the prediction accuracy of the CNN network for image weights in this way. The predicted weights also affect the learning of subsequent 3D LUTs parameters. Our method improves image enhancement by enhancing the weight prediction accuracy and the parameters of 3D LUTs.

(3) We conduct extensive experiments to compare our approach with some existing methods on one public dataset. The results demonstrate the superiority of our method in terms of performance, both data and visual effects.

## 2. Materials and Methods

### 2.1. Three-Dimensional Lookup Table

**Figure 1:**
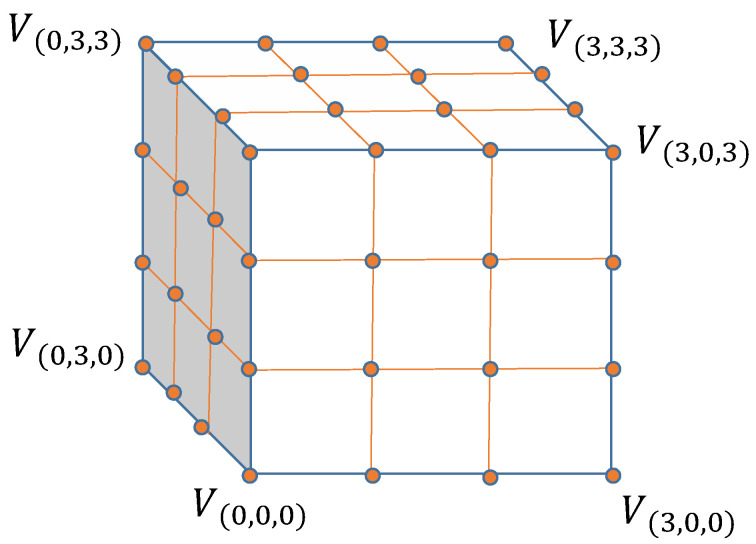
Illustration of a 3D LUT containing $4^{3}$ elements.

3D LUT is a classic and efficient image enhancement technique that has been widely and effectively used. As shown in Figure 1, a 3D LUT has three dimensional channels, representing the RGB color index. Each color channel is divided into *M* units so that a 3D LUT consists of $M^{3}$ elements ${\left\{V_{\left(i,j,k\right)}\right\}}_{i,j,k=0,\ldots ,M-1}$, where *M* is the number of units in any color channel. Each element $V_{\left(i,j,k\right)}$ defines an input $\left\{r_{\left(i,j,k\right)}^{I},g_{\left(i,j,k\right)}^{I},b_{\left(i,j,k\right)}^{I}\right\}$ of RGB value and the corresponding transformed output $\left\{r_{\left(i,j,k\right)}^{O},g_{\left(i,i,k\right)}^{O},b_{\left(i,j,k\right)}^{O}\right\}$. For a given *M*, the indexed color value ${\left\{r_{\left(i,j,k\right)}^{I},g_{\left(i,j,k\right)}^{I},b_{\left(i,j,k\right)}^{I}\right\}}_{i,j,k=0,\ldots ,M-1}$ can be obtained in a uniform color space. Different 3D LUTs will have different outputs so that different color outputs ${\left\{r_{\left(i,j,k\right)}^{O},g_{\left(i,j,k\right)}^{O},b_{\left(i,j,k\right)}^{O}\right\}}_{i,j,k=0,\ldots ,M-1}$ will be obtained after a given color index. The scale of *M* determines the accuracy of the color conversion. The larger the *M* is, the higher the accuracy of the color conversion will be. In our experiments, we set *M* = 33. In this case, a 3D LUT contains $108\;K\left(3M^{3}\right)$ parameters.

The 3D LUT transforms and transmits the color in two steps: lookup and trilinear interpolation. Firstly, given the input RGB color value $\left\{r_{\left(x,y,z\right)}^{I},g_{\left(x,y,z\right)}^{I},b_{\left(x,y,z\right)}^{I}\right\}$, the 3D LUT lattice is calculated to find its corresponding position (x,y,z). Here, *x*, *y*, *z* can be expressed as $x=\frac{r_{\left(x,y,z\right)}^{I}}{s},y=\frac{g_{\left(x,y,z\right)}^{I}}{s},z=\frac{b_{\left(x,y,z\right)}^{I}}{s}$, respectively, where $s=\frac{C_{\max}}{M}$, $C_{\max}$ is the maximum value on each color channel. After the position of the input color value is determined, trilinear interpolation can be performed using the eight elements closest to it to obtain the output RGB value. Let i=⌊x⌋,j=⌊y⌋,k=⌊z⌋, where ⌊.⌋ is the floor function, and let $d_{x}=\frac{r_{\left(x,y,z\right)}^{I}-r_{\left(i,j,k\right)}^{I}}{s}$, $d_{y}=\frac{g_{\left(x,y,z\right)}^{I}-g_{\left(i,j,k\right)}^{I}}{s}$, $d_{z}=\frac{b_{\left(x,y,z\right)}^{I}-b_{\left(i,j,k\right)}^{I}}{s}$. The output RGB $\left\{r_{\left(x,y,z\right)}^{O},g_{\left(x,y,z\right)}^{O},b_{\left(x,y,z\right)}^{O}\right\}$ after the color transformation will be obtained by trilinear interpolation and can be given by the following expression:

(1) $$\begin{array}{cc} c_{\left(x,y,z\right)}^{O} & =\left(1-d_{x}\right)\left(1-d_{y}\right)\left(1-d_{z}\right)c_{\left(i,j,k\right)}^{O}+d_{x}\left(1-d_{y}\right)\left(1-d_{z}\right)c_{\left(i+1,j,k\right)}^{O}\\ \; & +\left(1-d_{x}\right)d_{y}\left(1-d_{z}\right)c_{\left(i,j+1,k\right)}^{O}+\left(1-d_{x}\right)\left(1-d_{y}\right)d_{z}c_{\left(i,j,k+1\right)}^{O}\\ \; & +d_{x}d_{y}\left(1-d_{z}\right)c_{\left(i+1,j+1,k\right)}^{O}+\left(1-d_{x}\right)d_{y}d_{z}c_{\left(i,j+1,k+1\right)}^{O}\\ \; & +d_{x}\left(1-d_{y}\right)d_{z}c_{\left(i+1,j,k+1\right)}^{O}+d_{x}d_{y}d_{z}c_{\left(i+1,j+1,k+1\right)}^{O}, \end{array}$$

where c∈{r,g,b}. The above trilinear interpolation is sub-differentiable, and it is easy to derive the gradient of $c_{\left(i,j,k\right)}^{O}$. Since the trilinear interpolation of each input is independent of other pixels, this transformation is convenient for parallel computation.

### 2.2. Instance Normalization Adaptive Modulator (INAM)

Normalization is a method applied in the data preparation process when features in the data have different ranges to change the values of numeric columns in the data set using an identical scale. The advantages of normalization are as follows. (1) Normalization of each feature maintains the contribution of each feature when some features have higher values than others. This ensures that the network is unbiased. (2) The network activation distribution changes due to changes in network parameters during training. To improve training performance, we use normalization to reduce internal covariance. (3) Normalization makes the loss plane smoother because normalization constrains the size of the gradient more strictly. (4) Normalization makes the optimization faster because it disallows the weights to explode everywhere and limits them to a specific range. (5) Normalization helps the network to apply regularization.

The IN operation is to apply normalization to a specific image instance and normalize it in the HW dimension, which is initially used for image style transfer. The generated result mainly depends on an image instance, and the mean and variance of each feature map channel will affect the final image’s style. Therefore, the normalization of the entire batch and entire sample are unsuitable for image stylization, since only *H* or *W* dimension is normalized. Model convergence can be accelerated, and the independence between each image instance and channel (various features) is maintained. However, in the instance normalization process, the features’ standard deviation will be compressed, and the network’s ability to distinguish edge information will be reduced, which results in a decrease in the network’s performance. Inspired by Ref. [30], we propose the INAM that amplifies pixel bias by adaptively predicting modulation factors to solve this issue.

**Figure 2:**
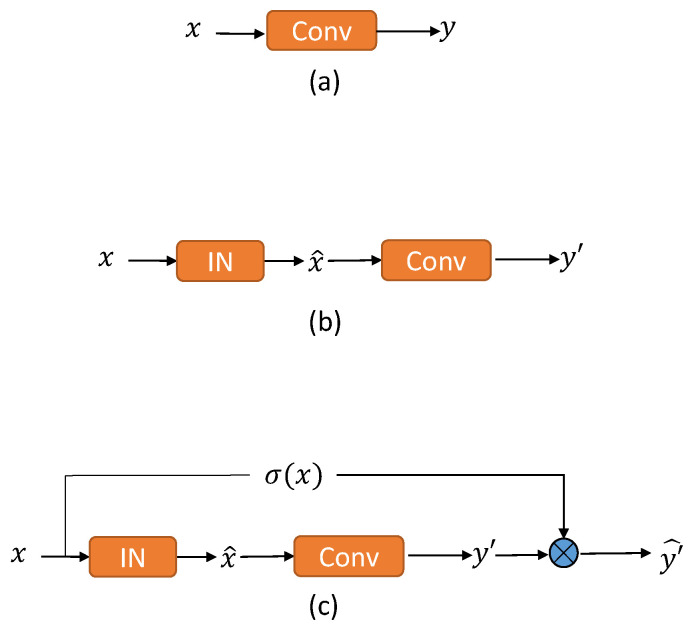
Three simple models for demonstration. (**a**) shows the model containing only one Conv layer without instance normalization. (**b**) shows the model containing a Conv layer and an instance normalization layer before Conv layer. (**c**) shows the model with an instance normalization layer, a Conv layer and a conditioning factor σ(x).

As shown in Figure 2, we constructed three simple models to demonstrate our approach. Figure 2a shows the model containing only one Conv layer without instance normalization. The input *x* is convolved to obtain the output *y*. Figure 2b shows the model constructed by inserting an IN layer before the Conv layer. The model shown in Figure 2c is constructed by inserting an IN layer before the Conv layer, i.e., the input *x* is first subjected to an instance normalization operation, and then the result of the operation is convolved to obtain the output *y*. The model shown in Figure 2c adds the IN layer after the input *x*, and then the output $y^{\prime }$ is obtained by convolution. $y^{\prime }$ is then multiplied by the conditioning factor to obtain the final output $\hat{y^{\prime }}$. For the sake of our exposition, we simplify this conditioning factor. We will describe the equations for these three models and then discuss the effects of feature normalization in terms of pixel standard deviation.

Denote the function of the Conv layer as $f_{Conv}$, the input of the Conv layer is *x*, and the output of the Conv layer is *y*. In Figure 2a, it is evident that we can describe the output *y* by the following formula:

(2) $$y=f_{Conv}\left(x\right)$$

In Figure 2b, *x* is a single input sample with four axes (*C*, *H*, *W*, *N*), and $\hat{x}$ is the transformed feature with IN. For IN, the mean and standard deviation are computed along the (*H*, *W*) axes so that we can compute $\hat{x}$ as:

(3) $$\hat{x}=\frac{x-\mu }{\sigma }$$

where μ and σ are scalars shared by all pixels in *x*, μ is the calculated mean of each instance, and σ is the calculated variance of all instances. Then the output $y^{\prime }$ can be computed by:

(4) $$\;y^{\prime }=f_{Conv}\left(\hat{x}\right)=f_{Conv}\left(\frac{x-\mu }{\sigma }\right)$$

Rewrite Equation (4) as:

(5) $$\;y^{\prime }=\frac{1}{\sigma }f_{\mathrm{Conv}}\left(x-\mu \right)$$

Equation (5) can be further expanded using the distributivity:

(6) $$\;y^{\prime }=\frac{1}{\sigma }f_{Conv}\left(x\right)-\frac{1}{\sigma }f_{Conv}\left(\mu I\right)$$

where *I* is the all-ones matrix with the same dimensions as *x*.

By comparing (2) and (6), it can be found that IN reshapes the pixel distribution of *y*. We compute the standard deviation (std) of $y^{\prime }$:

(7) $$\begin{array}{cc} std(y^{\prime }) & =std\left(\frac{1}{\sigma }f_{\mathrm{Conv}}\left(x\right)-\frac{1}{\sigma }f_{\mathrm{Conv}}\left(\mu I\right)\right)\\ \; & =\frac{1}{\sigma }std\left(f_{\mathrm{Conv}}\left(x\right)\right)\\ \; & =\frac{1}{\sigma }std\left(y\right). \end{array}$$

It can be seen that with instance normalization, the pixel bias is reduced to $\frac{1}{\sigma }$. The standard deviation decreases because σ is usually bigger than 1. To compensate for the loss of pixel bias, we multiply $y^{\prime }$ by σ in the third model, $\hat{\;y^{\prime }}$ can be obtained by:

(8) $$\hat{y^{\prime }}=\;y^{\prime }\cdot \sigma$$

### 2.3. INAM-Based Image-Adaptive 3D LUTs

Learning image-adaptive 3D LUTs is an effective color-mapping operation. Its process is divided into three steps, the first of which is using a weight predictor to predict the weight of the down-sampled low-resolution image and then using a look-up table generated from this weight for looking up and interpolation. For simplicity of description, we do not describe the interpolation operation in the 3D LUT but simplify it to look up in this subsection. Equation (9) represents a mapping function. In the RGB color domain, a classic 3D LUT is defined as a 3D cube containing $N^{3}$ elements, where *N* is the number of bins in each color channel. Each element defines a pixel-to-pixel mapping μ(x), where *x* is the input image, and $q_{o}$ is the output image.

(9) $$q_{o}=\mu \left(x\right)$$

**Figure 3:**
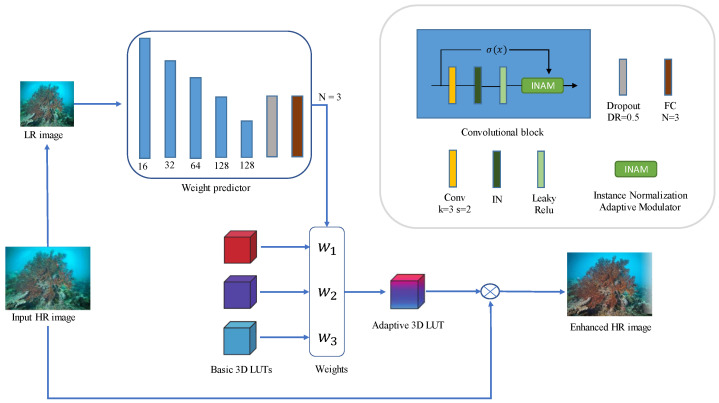
Overview of our proposed framework. The weight predictor outputs weights, and basic 3D LUTs form adaptive 3D LUTs based on the weights. The adaptive 3D LUT then enhances the input image. In the weight predictor, our proposed INAM is used.

However, the weight predictor used in learning image-adaptive 3D LUTs method to predict weights uses instance normalization (IN), which makes the standard deviation of the features compressed so that the network’s performance is reduced. That is, the model’s ability to distinguish edge information is reduced, resulting in a reduction in the accuracy of weight assignment. The INAM adaptively adjusts the feature standard deviation based on instance normalization, which improves the model’s ability to distinguish edge information and makes the weight prediction more accurate. The overall frame is shown in Figure 3.

**Figure 4:**
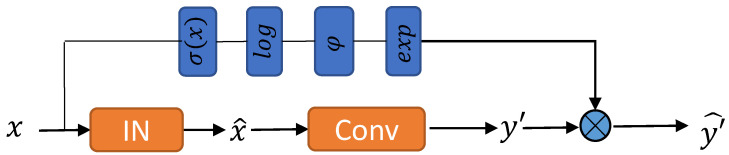
Illustration of INAM in the actual model.

As shown in Figure 3, the input of the model is a 480 × 640 high-resolution image, and the input of the weight predictor is a down-sampled low-resolution image with a resolution of 256 × 256. The weight predictor consists of five convolution blocks, a dropout layer and a fully connected layer. The output is the weight *N* which is set to 3 in our experiment. Each convolution block consists of a convolution layer, an IN layer, a leaky Relu layer, and an INAM. The first convolutional layer uses a three-channel image with an input of 256 × 256, a convolutional kernel of size 3 × 3, and an output of 16 channels. The second convolutional layer has an input size of 128 × 128 and an output of 32 channels. The third convolutional layer has an input size of 64 × 64 and outputs 64 channels. The fourth convolutional layer has an input size of 32 × 32 and outputs 128 channels. The fifth convolutional layer has an input size of 16 × 16 and outputs 128 channels. All the convolutional layers use a convolutional kernel size of 3 × 3, with a stride of 2 and padding of 1. The input size of the fully connected layer is 8 × 8, the convolutional kernel size is 8 × 8, and the number of output channels is N = 3. Based on the above data, the complexity of this CNN model can be calculated. The number of its parameters is about 191 k, and the number of FLOPs is 155 M. In Section 2.2, we introduced the basic principles of INAM through three simple models. However, in practical applications, the model will be more complex. For example, bias terms exist in each convolution, and there may be non-linear layers between convolutional layers. Equation (10) describes the INAM in the actual model:

(10) $$\hat{y^{\prime }}=\;y^{\prime }\cdot e^{\phi (\log(\sigma (x)))}$$

where *x* is the input, σ is the calculated standard deviation of *x*, *y* is the feature to be adjusted, $y^{\prime }$ is the output after modulating the feature, and ϕ(v):=w·v+b is a learnable linear model consisting of a weight *w* and a bias *b*. During training, *w* and *b* can be updated via the backpropagation algorithm. The ϕ function predicts an appropriate modulation factor based on the input value *v*. In Equation (10), we learn ϕ in logarithmic space for better stability. Finally, the modulation factor is obtained by exponential operation. The model is shown in Figure 4.

We learn several basic 3D LUTs and INAM-based CNN weight predictors. 3D LUTs are used to process the images and INAM-based CNNs are used to predict the weights of the images. Assuming that the weights obtained from the prediction are ${\left\{w_{n}\right\}}_{n=1,\ldots ,N}=f\left(x\right)$ and the corresponding 3D LUT processing is ${\left\{\mu_{n}\right\}}_{n=1,\ldots ,N}$, the final enhanced image obtained is:

(11) $$q=\sum_{n=1}^{N}w_{n}\mu_{n}\left(x\right)$$

where *x* indicates the input image. The objective function of our learning scheme can be written as follows:

(12) $$\min_{f,\mu_{n}}L\left(q,y\right)$$

where *f* and $\mu_{n}$ are the CNN model and the basic 3D LUTs to be learned, L(q,y) indicates some loss functions and regularization terms.

### 2.4. Loss Function

We use supervised learning methods to learn image enhancement models. Suppose that there are a number of *T* training pairs ${\left\{x_{t},y_{t}\right\}}_{t=1.2\ldots .T}$, where $x_{t}$ and $y_{t}$ denote a pair of input and target images, respectively. We employ the Mean Square Error (MSE) loss to train the model:

(13) $$L_{mse}=\frac{1}{T}\sum_{t=1}^{T}{∥q_{t}-y_{t}∥}^{2}$$

Using $L_{mse}$ loss, we train 3D LUTs with momentum or Adam optimizer and CNN weight predictors using the gradient descent algorithm. However, the optimized 3D LUTs may have unsmooth surfaces. The color mutations in the neighboring lattices of the 3D LUTs may amplify the chromatic aberration after color conversion, resulting in some banding artifacts in the smooth regions of the enhanced images. In order to make the learned 3D LUTs more stable and robust, we introduce two regularization terms in the optimization process.

Smoothing regularization: We introduce a 3D smoothing regularization term in 3D LUTs learning, which converts the input RGB values more stably into the desired color space, thus making the output of the 3D LUTs locally smooth. We choose the $L_{2}$ distance in the above term to achieve smoother regularization. To improve the smoothness of the adaptive 3D LUT, we introduce $L_{2}$-norm regularization for the prediction weights $w_{n}$. The overall smooth regularization term is as follows:

(14) $$\begin{array}{cc} R_{s} & =\sum_{c\in \{r,g,b\}}\sum_{i,j,k}\left(∥c_{\left(i+1,j,k\right)}^{O}-c_{\left(i,j,k\right)}^{O}{∥}^{2}+∥c_{\left(i,j+1,k\right)}^{O}\right.\\ \; & \left.-c_{\left(i,j,k\right)}^{O}{∥}^{2}+{∥c_{\left(i,j,k+1\right)}^{O}-c_{\left(i,j,k\right)}^{O}∥}^{2}\right)+\sum_{n}{∥w_{n}∥}^{2}. \end{array}$$

Monotonicity regularization: In addition to smoothness, 3D LUTs should also be monotonic. This is because monotonic transformations maintain the relative brightness and saturation of the input RGB values, ensuring natural enhancement results. Monotonicity helps update parameters that may not be activated by the input RGB values, improving the generalization ability of the learned 3D LUTs. Therefore, we adopt a monotonic regularization as follows:

(15) $$\begin{array}{cc} R_{m} & =\sum_{c\in \{r,g,b\}}\sum_{i,j,k}\left[g\left(c_{\left(i,j,k\right)}^{O}-c_{\left(i+1,j,k\right)}^{O}\right)+g\left(c_{\left(i,j,k\right)}^{O}\right.\right.\\ \; & \left.\left.-c_{\left(i,j+1,k\right)}^{O}\right)+g\left(c_{\left(i,j,k\right)}^{O}-c_{\left(i,j,k+1\right)}^{O}\right)\right] \end{array}$$

where g(·) is defined as the standard ReLU operation, i.e., g(a)=max(0,a). The monotonicity regularization ensures that the output RGB values $c_{\left(i,j,k\right)}^{O}$ increase with the index i,j,k and larger i,j,k indices correspond to larger input RGB values in the 3D LUT lattice.

By incorporating the two regularization terms, the final loss function used in learning is as follows:

(16) $$L=L_{\mathrm{mse}\;}+0.0001*R_{s}+10*R_{m}$$

### 2.5. Experimental Setup

#### 2.5.1. Dataset

We conduct experiments on the EUVP dataset. The dataset contains a large number of paired and unpaired underwater images with good or poor perceptual quality. These images are collected at different locations with different visibility conditions, and most of them are taken during ocean exploration and human-robot cooperation experiments. These images are carefully selected to accommodate a wide range of natural variations in the data. We use paired data of underwater-scenes from EUVP dataset, which contains 2185 raw underwater images covering different underwater scenes, underwater creatures, etc. We randomly select 2000 images as the training set and the remaining 185 images as the test set. We uniformly resize the image to 640 × 480 pixels for the experiment of 480 pixels.

#### 2.5.2. Baselines

We compare the perceptual image enhancement performance of the INAM-based image-adaptive 3D LUTs with the following models: (1) relative global histogram, unsupervised color correction (UCM) [12]; (2) contrast limited adaptive histogram equalization (CLAHE) [31]; (3) underwater dark channel prior (UDCP) [32]; (4) Water-net [33]; and (5) learning image-adaptive 3D LUTs [28]. The first three are physics-based models, and the Water-net is a learning-based model.

#### 2.5.3. Evaluation Metrics

In order to evaluate the image enhancement methods in a multifaceted way, four metrics are used for evaluation. They can be classified as full-reference evaluation metrics and no-reference evaluation metrics.

1. Full-Reference Evaluation:

PSNR is a Full-Reference image evaluation metric. It is one of the most common and widely used objective image evaluation metrics, which is based on the error between the corresponding pixel points, i.e., on error-sensitive image quality evaluation. A larger value indicates less distortion.

SSIM is a Full-Reference image evaluation index, which measures image similarity in brightness, contrast, and structure. Its value range is [0, 1]. A larger value indicates a smaller image distortion.

2. Non-Reference Evaluation:

UCIQE [34] is a linear combination of color intensity, saturation, and contrast, which is used to quantitatively evaluate the non-uniform color shift, blurring, and low contrast of underwater images. It is a Non-Reference (ground-truth) image quality evaluation index, and higher values indicate better image quality.

UIQM [35] is a Non-Reference underwater image quality evaluation index based on the stimulation of the human eye visual system, which adopts color, sharpness, and contrast measurements evaluation basis for the degradation mechanism and imaging characteristics of underwater images. A larger value means better color balance, sharpness, and contrast.

#### 2.5.4. Experiment Settings

Using the EUVP dataset, we experiment with color enhancement pipelines in camera imaging. In such applications, the target image is in the sRGB color space, has 8 bit of dynamic range, and is compressed to JPG format. In the photo retouching application, the input image has the same format as the target image. We learn the color enhancement part of the imaging pipeline instead of learning the entire pipeline from the raw data to the final RGB output. We use 2000 underwater images as the training set for image enhancement. One image per batch is selected with a size of 640 × 480. The model is trained using the ADAM optimizer, and the learning rate is set to $10^{-4}$ and reduces by half every 200 epochs. The model is trained for a total of 400 epochs. We implement our network with PyTorch and train all modules on an NVIDIA GTX1060ti GPU.

## 3. Results

### 3.1. Comparison Experiments

**Figure 5:**
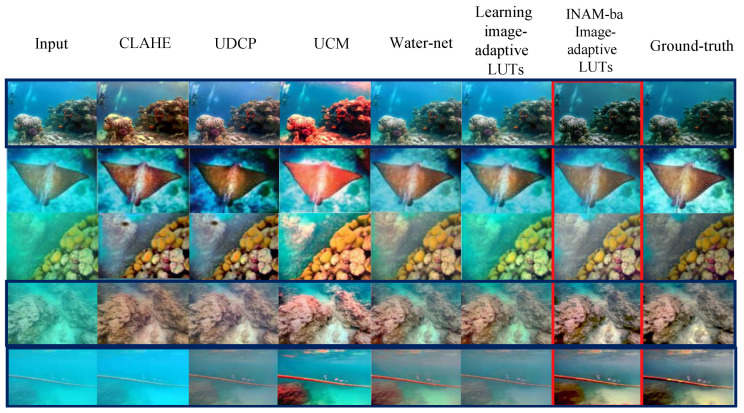
Qualitative performance comparison of UCM [12], CLAHE [31], UDCP [32], Water-net [33], learning image-adaptive 3D LUTs [28], and INAM-based image-adaptive 3D LUTs.

We first perform image enhancement using each method and compare the enhanced image with the ground-truth image using full-reference metrics to evaluate our method against the other methods. As shown in Table 1, our method outperforms UCM by 10.36 points or 71.39% in the PSNR metric. Our method is 6.54 points higher than CLAHE, which is 35.68% higher. Our method outperforms UDCP by 8.12 points or 48.48%. Our method outperforms Water-net by 4.83 points, or 24.10%. Compared to the original learning image-adaptive 3D LUTs method without adding the INAM module, our method improves by 3.85 points. This indicates that the pixel-to-pixel error between the image enhanced by our method and the ground-truth image is minimal. In the SSIM metric, our method is 0.390 points higher than UCM, which is 74.7% higher. It is 0.260 points or 39.87% higher than CLAHE, 0.358 points or 64.62% higher than UDCP, 0.209 points or 29.73% higher than the Water-net method, and 0.056 points or 6.5% higher than the original learning image-adaptive 3D LUTs method. This indicates that the enhanced image of our method is much better than other methods in all three aspects of brightness, contrast, and structure, which proves that our proposed INAM improves the 3D LUT method performance. An intuitive qualitative performance comparison is shown in Figure 5.

We perform image enhancement using each method and evaluate the enhanced images using the no-reference metrics UCIQE and UIQM. The obtained data are shown in Table 2. In the UCIQE metric, our method is 0.078 points higher than the UCM, which is 13.56% higher. Our method is 0.054 points or 9.01% higher than CLAHE. Our method is 0.068 points or 11.62% higher than UDCP. Our method outperforms Water-net by 0.047 points, or 7.76%. Compared with the learning image-adaptive 3D LUTs method without adding the INAM module, our method improves by 0.030 points, which is 4.8% higher. This indicates that the image quality enhanced by our method is the highest compared with other methods. In the UIQM metric, our method is 0.155 points or 11.27% higher than the UCM. It is 0.135 points higher than CLAHE, which is 9.64% higher. It is 0.120 points or 8.47% higher than UDCP, 0.181 points or 13.36% higher than the Water-net method, and 0.102 points or 7.11% higher than the original learning image-adaptive 3D LUTs method. This demonstrates that the enhanced image of our method is far better than other methods in three aspects: color balance, contrast, and sharpness structure, and it is more in line with human eye perception.

The INAM proposed by us has recovered the residual standard deviation reduced by IN through an operation. In this process, the network’s ability to recognize edge information is enhanced, and the prediction accuracy of weight is improved. This enables the model to learn excellent parameters in the learning process quickly. During the test, the image enhanced by the model will have better saturation, brightness contrast, and the highest structural similarity with the ground truth image.

### 3.2. Ablation Study

The number N of 3D LUTs: To verify the effect of the number of 3D LUTs on the image enhancement, we set the number of LUTs as 1, 2, 3, 4, 5. We still train the model on the EUVP dataset and evaluate the model to observe the effect of the number of LUTs on the image enhancement. We first conducted ablation experiments controlling the number of LUTs and obtained the data as shown in Table 3. From the data in the table, we can see that the values of PSNR, SSIM, UCIQE, and UIQM significantly increase as *N* increases from 1 to 3. While with *N* increasing from 3 to 5, the increase of these indicators is relatively small. Therefore, we use the number of LUTs as 3 in the comparison test.

**Figure 6:**
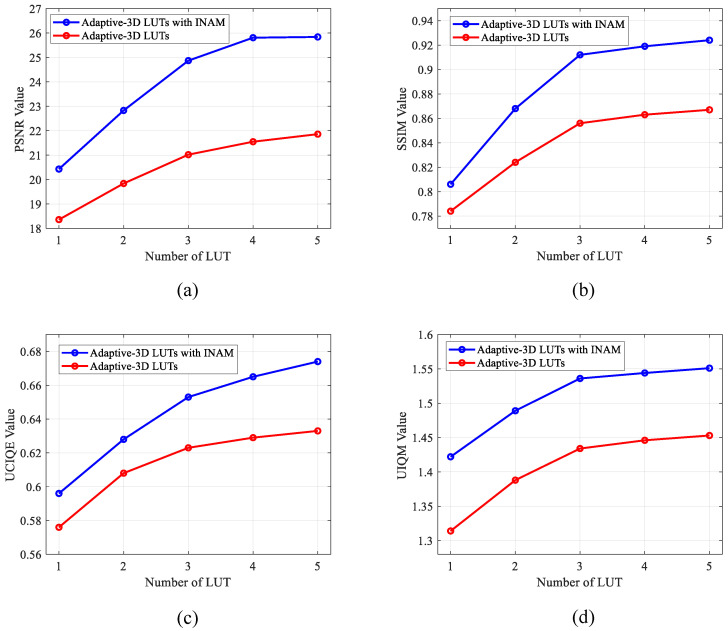
The performance of the learning image-adaptive 3D LUTs method and our proposed INAM-based image-adaptive 3D LUTs are compared in a line graph for each metric. The performance comparison of the two methods on PSNR metrics is shown in (**a**). (**b**) The comparison of the two methods in terms of SSIM performance. (**c**) Comparison of the performance of the two methods on the UCIQE metric. A comparison of the two methods evaluated by the UIQM metrics is shown in (**d**).

INAM: To verify the effectiveness of our proposed INAM, we set the number of LUTs in the learning image-adaptive 3D LUTs method without adding the INAM module, and train the model on the EUVP dataset and evaluate it. The data are compared with the INAM-based image-adaptive 3D LUTs method to verify the effectiveness of our proposed INAM module for image enhancement. We change the number of LUTs for the learning image-adaptive 3D LUTs method without the INAM and obtain the data in Table 4. We compared it with the INAM-based image-adaptive 3D LUTs method at the same number of LUTs. The comparison results are shown in Figure 6. Our method outperforms the learning image-adaptive 3D LUTs method without INAM for different N in PSNR, SSIM, UCIQE, and UIQM metrics. This comparison verifies the effectiveness of our proposed INAM.

## 4. Conclusions

We introduced the INAM into learning image-adaptive 3D LUTs for underwater image enhancement. After instance normalization, the standard deviation of features will be reduced, which reduces the ability of the network to distinguish edge information and thus, the accuracy of weight assignment will be reduced. Our proposed INAM can compensate for the compressed standard deviation and thus improve the accuracy of the weight predictor. We train and test on the EUVP dataset and evaluate its effectiveness by comparing with other traditional and learning-based methods. The experimental results show that the method outperforms other methods in PSNR, SSIM, UCIQE, and UIQM metrics. We conducted ablation experiments to verify the effect of the number of LUTs on our proposed method. We also verified the performance improvement of our proposed INAM on the image-adaptive 3D LUTs method for different numbers of LUTs.

## 5. Discussion

In this study, we propose INAM, which can compensate for the change of standard deviation caused by instance normalization through learning. We apply it in the weight prediction module of the adaptive 3D LUT, which significantly improves the underwater image enhancement effect in all indicators without affecting the training speed and generalization accuracy.

We consider the CNN module to be necessary in the image enhancement process. This is because the fraction of LUT weights it predicts directly determines the size of this LUT’s role in the subsequent image enhancement process. In past CNN modules that used IN, the standard deviation of the residual features would be greatly reduced. The standard interpolation of the residual features would reflect the variation of the pixel values. We propose the INAM module, which allows CNN to improve the standard deviation of the residual features while maintaining the training speed and generalization accuracy. This operation enables the CNN module to improve the recognition of edges and allows the prediction accuracy of weights to be improved when the CNN and 3D LUTs are trained together. This allows for better coordination between several 3D LUTs in the processing of images and also impacts the parameters being learned by the 3D LUTs. Therefore, our proposed method has an excellent performance in all metrics.

Our proposed INAM can improve the edge recognition ability of CNN by amplifying the pixel bias through adaptive prediction of modulation factors. It may be helpful for other networks, such as edge detection, semantic segmentation, and other tasks.

## Figures and Tables

**Table 1:** Quantitative comparison for average PSNR and SSIM values on test images of the EUVP dataset.

Model	PSNR	SSIM
UCM	14.51	0.522
CLAHE	18.33	0.652
UDCP	16.75	0.554
Water-net	20.04	0.703
Learning image-adaptive 3D LUTs	21.02	0.856
**INAM-based image-adaptive 3D LUTs**	**24.87**	**0.912**

**Table 2:** Quantitative comparison for average UCIQE and UIQM values on test images of the EUVP dataset.

Model	UCIQE	UIQM
UCM	0.575	1.375
CLAHE	0.599	1.401
UDCP	0.585	1.416
Water-net	0.606	1.355
Learning image-adaptive 3D LUTs	0.623	1.434
**INAM-based image-adaptive 3D LUTs**	**0.653**	**1.536**

**Table 3:** Ablation studies on the number (N) of LUTs affecting the INAM-based image-adaptive 3D LUTs.

N	1	2	3	4	5
PSNR	20.43	22.83	24.87	25.81	25.84
SSIM	0.806	0.868	0.912	0.919	0.924
UCIQE	0.596	0.628	0.653	0.665	0.671
UIQM	1.422	1.489	1.536	1.544	1.551

**Table 4:** Ablation studies on the number (N) of LUTs affecting the image-adaptive-3D LUTs without INAM.

N	1	2	3	4	5
PSNR	18.36	19.84	21.02	21.55	21.86
SSIM	0.784	0.824	0.856	0.863	0.867
UCIQE	0.576	0.608	0.623	0.629	0.633
UIQM	1.314	1.388	1.434	1.446	1.453

## Data Availability

The data used in this work is the EUVP dataset. It can be download from https://irvlab.cs.umn.edu/resources/euvp-dataset (accessed on 15 December 2022).
